# The Cultivation Method Affects the Transcriptomic Response of Aspergillus niger to Growth on Sugar Beet Pulp

**DOI:** 10.1128/spectrum.01064-21

**Published:** 2021-08-25

**Authors:** Sandra Garrigues, Roland S. Kun, Mao Peng, Birgit S. Gruben, Isabelle Benoit Gelber, Miia Mäkelä, Ronald P. de Vries

**Affiliations:** a Fungal Physiology, Westerdijk Fungal Biodiversity Institute & Fungal Molecular Physiology, Utrecht University, Utrecht, The Netherlands; b Microbiology, Utrecht University, Utrecht, The Netherlands; c Department of Microbiology, University of Helsinki, Helsinki, Finland; Broad Institute

**Keywords:** gene expression, submerged culture, solid culture, *Aspergillus niger*, sugar beet pulp

## Abstract

In nature, filamentous fungi are exposed to diverse nutritional sources and changes in substrate availability. Conversely, in submerged cultures, mycelia are continuously exposed to the existing substrates, which are depleted over time. Submerged cultures are the preferred choice for experimental setups in laboratory and industry and are often used for understanding the physiology of fungi. However, to what extent the cultivation method affects fungal physiology, with respect to utilization of natural substrates, has not been addressed in detail. Here, we compared the transcriptomic responses of Aspergillus niger grown in submerged culture and solid culture, both containing sugar beet pulp (SBP) as a carbon source. The results showed that expression of CAZy (Carbohydrate Active enZyme)-encoding and sugar catabolic genes in liquid SBP was time dependent. Moreover, additional components of SBP delayed the A. niger response to the degradation of pectin present in SBP. In addition, we demonstrated that liquid cultures induced wider transcriptome variability than solid cultures. Although there was a correlation regarding sugar metabolic gene expression patterns between liquid and solid cultures, it decreased in the case of CAZyme-encoding genes. In conclusion, the transcriptomic response of A. niger to SBP is influenced by the culturing method, limiting the value of liquid cultures for understanding the behavior of fungi in natural habitats.

**IMPORTANCE** Understanding the interaction between filamentous fungi and their natural and biotechnological environments has been of great interest for the scientific community. Submerged cultures are preferred over solid cultures at a laboratory scale to study the natural response of fungi to different stimuli found in nature (e.g., carbon/nitrogen sources, pH). However, whether and to what extent submerged cultures introduce variation in the physiology of fungi during growth on plant biomass have not been studied in detail. In this study, we compared the transcriptomic responses of Aspergillus niger to growth on liquid and solid cultures containing sugar beet pulp (a by-product of the sugar industry) as a carbon source. We demonstrate that the transcriptomic response of A. niger was highly affected by the culture condition, since the transcriptomic response obtained in a liquid environment could not fully explain the behavior of the fungus in a solid environment. This could partially explain the differences often observed between the phenotypes on plates compared to liquid cultures.

## INTRODUCTION

Filamentous fungi are widely recognized for their ability to produce large amounts of metabolites, organic compounds, and enzymes for many industrial applications, such as food and feed, pulp and paper, textiles, detergents, and biofuel and biochemicals (1, 2). Fungi show several advantages for protein production over other microorganisms, such as bacteria, since high-level secretion of enzymes is a common trait of their decomposer lifestyle. However, the type and morphology of growth have critical implications for gene expression, affecting the resulting production of enzymes and metabolites, and these are major aspects of fungal biology that are not yet comprehensively understood (3). Filamentous fungi are ubiquitous organisms that can colonize almost all ecosystems on earth. In their natural habitat, different parts of the mycelium are exposed to heterogeneous nutritional sources with variable compositions. In solid cultures, fungal colonies are exposed to changes in the available substrates during the course of hyphal extension. While the colony center is in contact with carbon sources that have been (partially) utilized, the periphery encounters unexplored organic compounds (4). In contrast, in submerged cultures, mycelia are exposed to more uniform substrates, which are utilized over time, and several mycelial forms can arise, from dispersed hyphae to compact pellets. Additionally, large pellets have been reported to have low metabolism and growth rates due to poor oxygen diffusion (3). Previous studies already demonstrated that protein production varies significantly between solid and submerged fermentation conditions. Aspergillus terreus improved cellulase production up to 14.6-fold during solid fermentation, where growth more closely resembled the natural growth conditions of this fungus (5). In Aspergillus brasiliensis, solid fermentation also increased secretome complexity compared to submerged cultures (6). In contrast, the basidiomycete Phanerochaete chrysosporium produced more carbohydrate-binding module-containing enzymes in submerged cultures compared to solid-state cultures (7).

Apart from the differences associated with growth type and conditions, previous studies also demonstrated that both gene expression and enzyme production can differ within different parts of the hyphae. For example, in Aspergillus niger, it was shown that while carbon catabolism-related genes were expressed uniformly during growth on solid sugar beet pulp (SBP) cultures, plant biomass-degrading enzyme-encoding genes were differentially expressed throughout the colony (8). In addition, in maltose- and xylose-grown colonies, more than 25% of the active genes differed significantly between the inner and outer parts of the colony (4), indicating a high degree of differentiation in fungal vegetative mycelia.

Understanding the interaction between fungi and their environments has been a topic of great interest for many decades, and postgenomic approaches, such as transcriptomics and proteomics, have enabled the discovery of new and detailed insights (9). Over the last few years, an increasing number of studies have aimed to understand the fungal responses to different environments and conditions. In this sense, there are many published studies, including some of our own, in which results obtained from liquid cultures are interpreted in the context of natural behavior/evolution of a fungus, despite the fact that the natural environment for most fungi better resembles solid cultures (10–16). Submerged cultures are the preferred choice for most experimental setups. However, whether submerged cultures resemble the natural growth conditions of fungi has not been addressed so far.

In this study, we aimed to reveal to what extent the A. niger responses to a plant biomass substrate are comparable when grown on solid and submerged cultures. For this, we first studied the gene expression pattern of A. niger grown in liquid SBP, a by-product of the sugar industry that consists mainly of pectin, cellulose, and xyloglucan (17). Furthermore, we compared the expression of sugar metabolic and Carbohydrate Active enZyme (CAZy)-encoding genes when grown in liquid SBP to that of the polygalacturonic acid (PGA) culture, which resembles a major component of the crude substrate. Finally, we compared the A. niger genetic response to liquid SBP culture to that of the same fungus grown on solid SBP plates (8).

## RESULTS AND DISCUSSION

### A. niger shows temporal adaptation to the degradation of sugar beet pulp.

Sugar beet is an underground plant that is grown commercially for sugar production. Sugar beet pulp (SBP) is a low-priced by-product of the sugar and ethanol industries that is traditionally used in the animal feed sector and constitutes an underexploited resource for many industrial applications due to its high sugar composition (see Table S1 in the supplemental material). Utilization of SBP as a complex substrate requires a diverse array of plant cell wall-degrading enzymes. Filamentous fungi produce a wide range of hydrolytic and oxidative enzymes that allow them to grow on complex and diverse types of plant biomass, including SBP (18).

**FIG 1:**
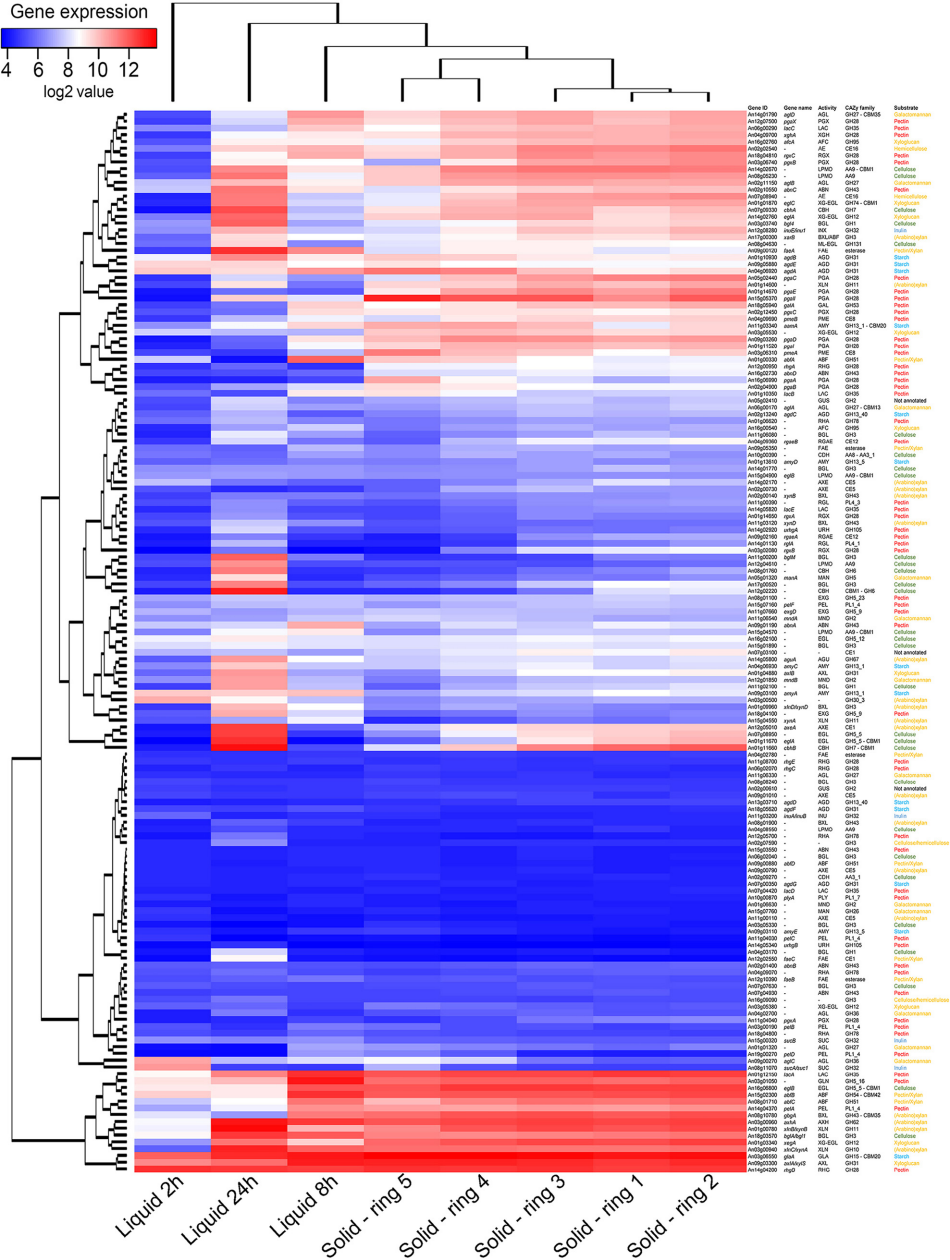
Hierarchical clustering of CAZyme-encoding genes in A. niger grown on 1% sugar beet pulp solid or submerged culture. Gene expression data originate from 2-, 8-, and 24-h liquid cultures and from five different stages of growth on solid medium (8). The substrates associated with the corresponding genes are indicated by different color codes (yellow for hemicellulose, red for pectin, green for cellulose, blue for storage polysaccharides, and black for undetermined substrates). Enzyme activity abbreviations are described in Table S2. Genes with an expression level of <20 across all samples were excluded from the analysis.

**FIG 2:**
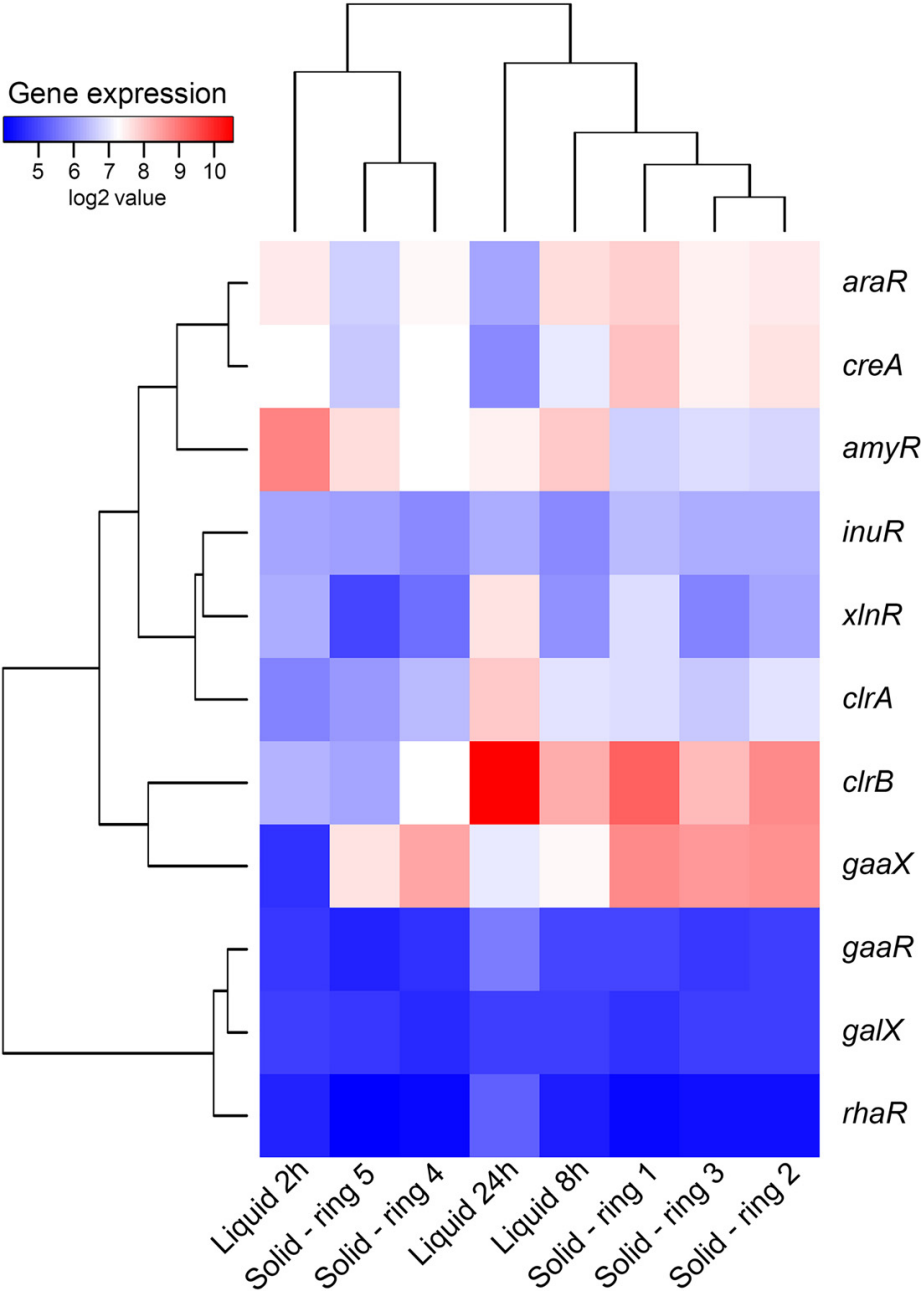
Hierarchical clustering of transcription factor-encoding genes in A. niger grown on 1% sugar beet pulp solid or submerged culture. Gene expression data originate from 2-, 8-, and 24-h liquid cultures and from five different stages of growth on solid medium (8).

Expression of genes encoding plant polysaccharide-degrading enzymes in A. niger grown in liquid SBP showed a significant change over time (Fig. 1, Supplementary Data Set S1), suggesting a sequential use of several SBP components. After 2 h of growth, the expression of Carbohydrate Active enZyme (CAZy; http://www.cazy.org) (19) encoding genes was generally low, which would indicate that (i) A. niger requires more time to induce CAZy genes (Fig. 1), or (ii) the presence of sucrose and free monosaccharides would prevent the expression of CAZy-encoding genes at early time points. Some exceptions were genes involved in starch (e.g., *amyA*, *glaA*, *agdA*, *agdE*) or sucrose (e.g., *sucA*) degradation. Starch, which is a polymeric carbohydrate consisting of d-glucose units linked by glycosidic bonds, is a predominant storage polysaccharide in underground plants. However, the sugar beet is an exception, since it exclusively stores sucrose, which is a disaccharide composed of d-glucose and d-fructose (20). The high expression of the invertase-encoding gene *sucA* at this early time point may suggest a preference of A. niger to utilize sucrose over other components of SBP. However, the high expression of starch-degrading enzyme-encoding genes in A. niger at this time point cannot be attributed to the presence of starch in SBP. This, however, could rather be explained by the presence of residual free d-glucose, which would be sufficient to induce starch-degrading genes as previously shown (12). Additionally, early release of d-glucose from sucrose or other SBP components could also explain the early induction of starch-degrading genes. These results also correlate with the high expression of the amylolytic transcription factor gene *amyR* at this time point (Fig. 2, Supplementary Data Set S2). AmyR, which is the main activator of genes involved in starch degradation, has been demonstrated to be induced in the presence of d-glucose in Aspergillus (21, 22).

A significant increase in the expression of pectinolytic genes (e.g., *abfA*, *afbB*, *abfC*, An03g01050, *pgaX*) and hemicellulolytic genes (e.g., *axlA*, *axhA*, *gbgA*, *aglD*) was detected after 8 h of growth in liquid SBP (Fig. 1). These results may indicate that A. niger preferentially uses pectin and hemicellulose after (partial) depletion of mono- and small oligosaccharides.

Cellulolytic genes (e.g., *cbhA*, *cbhB*, *bgl4*, *bglM*, *eglA*) were expressed at a low level after 2 or 8 h of incubation. However, these genes showed their highest expression after 24 h of growth (Fig. 1), correlating with a decrease in expression of pectinolytic genes. This also correlates with the expression levels of the genes encoding the transcription factors involved in cellulose degradation (*xlnR*, *clrA*, *clrB*) (23, 24), which were highly induced after 24 h of growth in liquid SBP (Fig. 2, Supplementary Data Set S2). These results indicate that cellulose is not a preferred carbon source for A. niger and is only degraded and utilized at later stages of growth in liquid SBP when other carbon sources such as pectin are (almost) depleted. Preferential use of substrate components by A. niger was also reported during growth on wheat bran, which mainly consists of cellulose, starch, and hemicellulose, and where a preference for (arabino)xylan was observed after 24 h of growth (24). Also in this study, cellulose was a nonpreferred carbon source of A. niger. This confirms the high flexibility of A. niger to adapt to the available carbon source depending on the substrate it grows on.

### The expression of sugar catabolic genes in A. niger grown in liquid sugar beet pulp correlates with the expression of CAZyme-encoding genes.

Analysis of expression of crucial genes involved in sugar catabolism of A. niger grown in liquid medium with SBP was performed to better understand the transcriptomic response of this fungus to this complex substrate.

**FIG 3:**
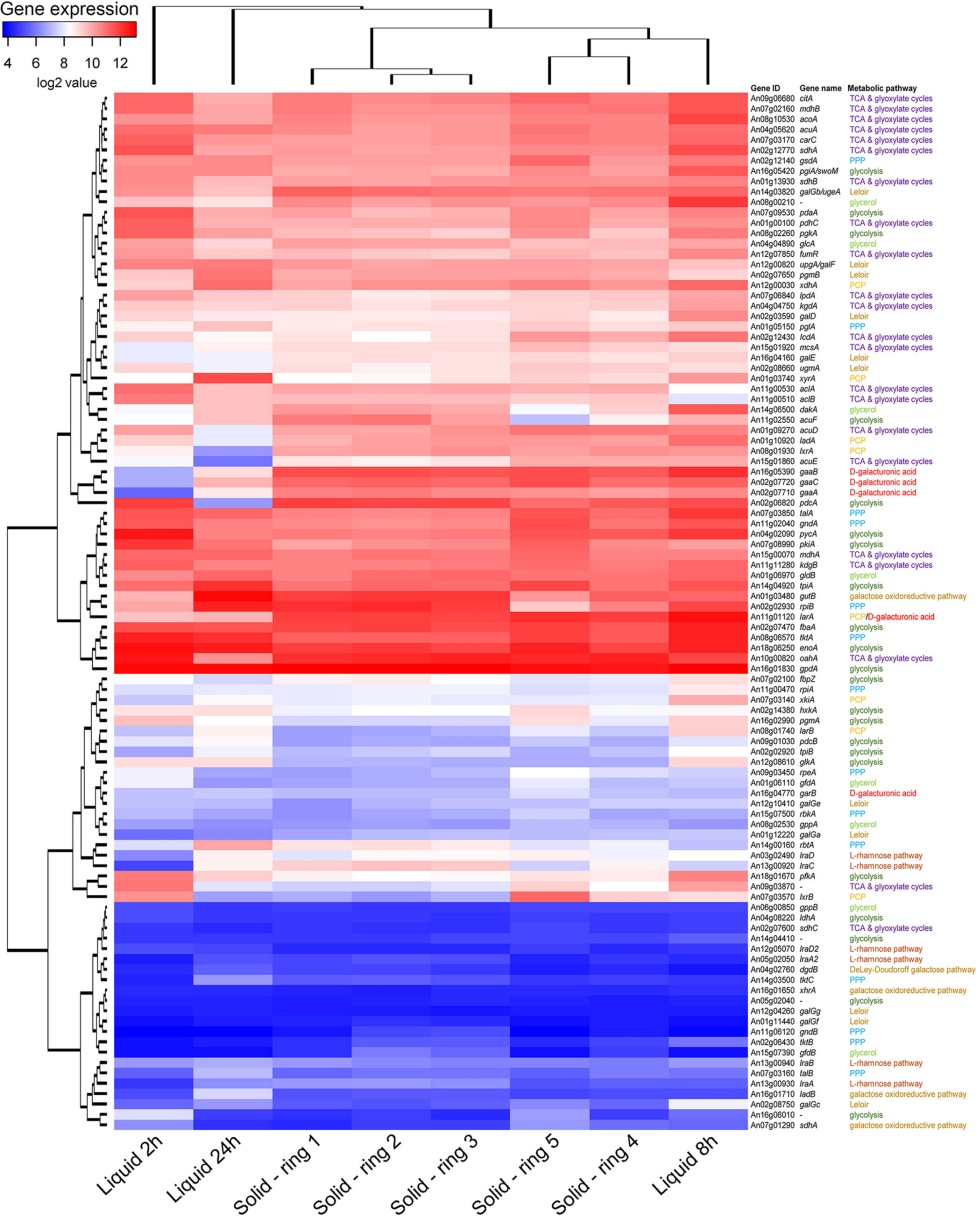
Hierarchical clustering of sugar-related metabolic genes in A. niger grown on 1% sugar beet pulp solid or submerged culture. Gene expression data originate from 2-, 8-, and 24-h liquid cultures and from five different stages of growth on solid medium (8). The metabolic pathways associated with the corresponding genes are indicated by different color codes (purple for the tricarboxylic acid [TCA] and glyoxylate cycles, blue for the pentose phosphate pathway [PPP], yellow for the pentose catabolic pathway [PCP], red for the d-galacturonic acid pathway, orange for the l-rhamnose pathway, gold for the d-galactose pathways, dark green for glycolysis, and light green for the glycerol pathway). Enzyme activities are indicated in Data Set S3. Genes with an expression level of <20 across all samples were excluded from the analysis.

Glycolysis is a cytoplasmic pathway that converts d-glucose molecules into pyruvate. After glycolysis, the end-compounds enter the tricarboxylic acid (TCA) cycle and energy is obtained. SBP is very rich in d-glucose (Table S1). It contains around 30 to 40% cellulose (17), a polysaccharide consisting of a linear chain of β(1, 4)-linked d-glucose units, and accumulates sucrose as a storage disaccharide (20). In general, genes involved in glycolysis and the TCA cycle were highly expressed at all time points (Fig. 3), with a slight decrease after 24 h of growth. This high expression from early time points can be explained by the preferred utilization of sucrose, as also suggested by the high expression of the extracellular invertase-encoding gene (*sucA*). Additionally, these results might also correlate with d-glucose release from the cellulose present in SBP and highlight the relevance of these pathways for energy generation in A. niger.

Sugar pentoses (e.g., d-xylose and l-arabinose) released from SBP are metabolized through the pentose catabolic pathway (PCP) and subsequently the pentose phosphate pathway (PPP) (25). After 2 h of submerged growth, genes involved in the l-arabinose-specific steps of PCP (e.g., *ladA*, *lxrA*, *lxrB*) were highly expressed (Fig. 3). These genes are mainly under the control of the transcriptional regulator AraR (26), which was also highly expressed under this condition (Fig. 2). Additionally, this pathway remained active after 8 and 24 h of growth in liquid SBP, while many other PCP genes were highly expressed at these time points (e.g., *xdhA*, *xyrA*, *xkiA*, *xyrB*) (Fig. 3). This pattern correlates with the increased expression of arabinofuranosidase-encoding genes (e.g., *abfA*, *afbB*, *abfC*) at 8 h (Fig. 1), which catalyze l-arabinose release from pectin side chains and are also under the control of AraR. PCP genes such as *xyrA*, *xkiA*, and *xdhA* are also regulated by the transcriptional regulator XlnR (26). These genes were highly expressed after 24 h of growth in liquid SBP, correlating with the high expression of this regulator at the same time point (Fig. 2). Similar to the PCP, PPP genes also have diverse temporal expression profiles. For example, the *gsdA*, *pglA*, *talA*, *gndA*, *tktA*, and *rpiB* genes were expressed at high levels at 2, 8, and 24 h, while other genes, such as *gndB*, *tktB* and *talB*, were expressed at low levels all time points (Fig. 3). A previous study showed that *tktB* and *gndB* presented minimal expression values when A. niger was cultivated in either d-xylose or l-arabinose (26). However, the expression of *talB* was substantially higher in d-xylose and l-arabinose than in SBP, but significantly lower than that of *talA*. Thus, the low expression of *gndB*, *tktB*, and *talB* could be explained by gene redundancy with respect to *gndA*, *tktA*, or *talA*, respectively.

The pectin backbone contains d-galacturonic acid as the main constituent, but pectins can also contain l-rhamnose residues, as well as l-arabinose and/or d-galactose mainly in the side chains (27). In contrast to the genes involved in the PCP, PPP, glycolysis, or TCA cycle, the genes involved in d-galacturonic acid catabolism were not expressed at all time points. Expression levels of the genes *gaaA*, *gaaB*, and *gaaC* were very low after 2 h of growth in liquid SBP. However, these genes were highly induced after 8 h of submerged growth in this crude substrate, and expression decreased after 24 h (Fig. 3). The expression of the transcriptional regulator GaaR, which activates genes involved in d-galacturonic acid release and utilization in A. niger (28), remained low at all time points, although its expression started to slightly increase at 8 h (Fig. 2), which correlated with the induction of the aforementioned d-galacturonic acid catabolic genes and showed the highest expression at 24 h. In contrast, the gene coding for the d-galacturonic acid transcriptional repressor GaaX (29) showed a strong induction after 8 h of growth in liquid SBP cultures (Fig. 2), which could be a response to the induction of the GaaR-encoding gene, which started at this time point. The expression of *gaaX* was still high after 24 h of growth (Fig. 2), correlating with the decreased expression of the d-galacturonic acid catabolic genes *gaaA*, *gaaB*, and *gaaC* at this time point.

Similarly, l-rhamnose catabolic genes (*lraA*, *lrdA*, and *lkaA*) showed relatively low expression after 2 h, and induction started at a later stage of growth, with the highest expression values after 24 h. These results correlate with the highest expression values after 24 h of the gene encoding the transcriptional activator RhaR, which is involved in the activation of genes responsible for l-rhamnose release and utilization (30). These results also correlate with the high expression of pectinolytic genes after 8 h of growth (Fig. 1).

On the other hand, genes involved in the d-galactose catabolic pathways (e.g., *galD*, *galF*, *galG*, *pgmB*) were overall highly expressed, which correlates with fast depletion of this monosaccharide in the culture medium (31).

### The complex composition of sugar beet pulp causes delayed pectin degradation in A. niger.

Pectin accounts for up to 30% of SBP composition (17) and has a high content of polygalacturonic acid (PGA). In this study, we evaluated the effect of additional nonpectin components of SBP on pectin degradation and assessed A. niger response to pectin degradation when growing in liquid medium with PGA or SBP as the sole carbon source. The gene expression profiles of A. niger cultured on 1% SBP and 1% PGA were compared after 2, 8, and 24 h of growth. Principal-component analysis (PCA) of duplicate samples showed that the A. niger response to SBP and to PGA is highly diverse (see Fig. S1 in the supplemental material). Gene expression in 2-h SBP and 2-h PGA samples showed the most differential patterns, whereas 24-h SBP and 24-h PGA samples showed the highest similarities. These results might indicate a delayed response in pectin degradation when A. niger is grown on SBP due to the other components of this substrate (Table S1). In general, the PGA sample showed higher expression of CAZy genes (including pectinolytic genes) (Table 1; see Fig. S2 in the supplemental material) and the main carbon metabolic pathway genes (Table 2) after 2 h of growth. Regarding CAZy-encoding genes, *abfA*, *abfB*, and *abfC*, which code for arabinofuranosidases involved in the release of l-arabinose units from pectic substances, were highest expressed in the PGA culture at 2 h. The high expression of these genes indicates that arabinofuranosidases play a crucial role at the initial stages of pectin degradation. In contrast, these genes showed significantly lower expression at 2 h in SBP and showed the highest expression after 8 h in this crude substrate (Table 1), which reflects a delayed pectin degradation by the fungus in SBP compared to PGA. Overall, 32 genes involved in pectin degradation were downregulated (<0.5-fold change), and only one was upregulated (*rhgB*) (>2-fold change) in the SBP culture compared to the PGA culture after 2 h of growth. Interestingly, not only pectinolytic genes, but also major (hemi)cellulolytic and several amylolytic genes, together with the exo-inulinase-encoding gene *inuE*, showed a higher expression level on PGA than on SBP, accounting for a total of 76 upregulated and only seven downregulated CAZy genes. This might be due to impurities found in PGA, which can induce the expression of a broad set of genes at an early stage of growth (12). Moreover, the expression of genes involved in d-galacturonic acid (*gaaA*, *gaaB*, *gaaC*), l-rhamnose (*lraA*, *lrlA*, *lrdA*, *lkaA*), or l-arabinose and d-xylose (*larA*, *xyrB*, *ladA*, *lxrA*, *xyrA*, *xdhA*, *xkiA*) catabolism showed significantly lower expression in the 2-h SBP culture (Table 2), which can be associated with a reduced ability to degrade pectin components.

**TABLE 1:** Comparative analysis of the expression of CAZyme-encoding genes involved in pectin degradation in A. niger when grown in liquid cultures with 1% sugar beet pulp or 1% polygalacturonic acid

Gene ID	Gene name	Activity	CAZy family	Expression with growth on:						Fold change in expression^*a*^		
				PGA			SBP					
				2 h	8 h	24 h	2 h	8 h	24 h	2 h	8 h	24 h
An12g07500	*pgaX*	PGX (exo-polygalacturonase)	GH28	5,084.05	1,685.30	2,729.56	29.61	1,338.03	230.83	0.01	0.79	0.08
An02g12450	*pgxC*	PGX (exo-polygalacturonase)	GH28	5,069.35	300.78	737.73	37.77	688.91	58.80	0.01	**2.29**	0.08
An03g06740	*pgxB*	PGX (exo-polygalacturonase)	GH28	4,073.59	1,851.40	8,247.77	38.70	416.64	499.93	0.01	0.23	0.06
An18g04810	*rgxC*	RGX (exo-rhamnogalacturonase)	GH28	2,769.18	183.66	1,166.60	30.12	1,292.78	469.07	0.01	**7.04**	0.40
An01g10350	*lacB*	LAC (β-1,4-galactosidase)	GH35	3,980.22	56.05	145.49	56.35	566.39	34.65	0.01	**10.11**	0.24
An09g03260	*pgaD*	PGA (endo-polygalacturonase)	GH28	975.72	254.53	90.29	15.40	587.06	50.82	0.02	**2.31**	0.56
An08g01710	*abfC*	ABF (α-arabinofuranosidase)	GH51	12,115.25	337.20	1,059.35	197.94	2,813.09	432.03	0.02	**8.34**	0.41
An01g00330	*abfA*	ABF (α-arabinofuranosidase)	GH51	10,130.04	28.94	69.17	216.77	3,659.83	14.77	0.02	**126.45**	0.21
An09g01190	*abnA*	ABN (endo-arabinanase)	GH43	8,146.15	67.85	359.03	193.12	1,240.63	231.69	0.02	**18.28**	0.65
An14g02920	*urhgA*	URH (unsaturated rhamnogalacturonyl hydrolase)	GH105	898.80	32.13	48.48	21.87	37.69	237.24	0.02	1.17	**4.89**
An01g11520	*pgaI*	PGA (endo-polygalacturonase)	GH28	688.71	167.16	73.95	19.51	365.71	46.83	0.03	**2.19**	0.63
An06g00290	*lacC*	LAC (β-1,4-galactosidase)	GH35	2,486.88	199.91	235.74	87.07	872.64	160.26	0.04	**4.37**	0.68
An04g09700	*xghA*	XGH (xylogalacturonase)	GH28	726.39	35.55	46.75	27.49	533.32	368.96	0.04	**15.00**	**7.89**
An01g12150	*lacA*	LAC (β-1,4-galactosidase)	GH35	9,933.46	528.61	2,525.83	457.88	6,789.32	2,198.62	0.05	**12.84**	0.87
An09g00120	*faeA*	FAE (feruloyl esterase)	Esterase	521.48	19.16	29.49	28.27	1,975.63	6,765.29	0.05	**103.09**	**229.40**
An15g02300	*abfB*	ABF (α-arabinofuranosidase)	GH54 -CBM42	13,314.99	125.96	1,621.92	820.73	6,795.91	580.38	0.06	**53.95**	0.36
An18g05940	*galA*	GAL (β-1,4-endo-galactanase)	GH53	440.12	112.50	260.78	29.04	811.80	60.28	0.07	**7.22**	0.23
An14g04370	*pelA*	PEL (pectin lyase)	PL1_4	1,783.12	1,445.63	964.70	119.25	1,888.09	331.77	0.07	1.31	0.34
An04g09360	*rgaeB*	RGAE (rhamnogalacturonan acetyl esterase)	CE12	362.35	106.51	347.33	27.15	61.51	226.93	0.07	0.58	0.65
An04g09690	*pmeB*	PME (pectin methyl esterase)	CE8	295.77	42.95	77.89	25.78	352.83	161.53	0.09	**8.21**	**2.07**
An03g01050		GLN (exo-1,6-galactanase)	GH5_16	7,041.39	152.41	177.36	624.32	10,010.03	1,052.80	0.09	**65.68**	**5.94**
An11g00390		RGL (rhamnogalacturonan lyase)	PL4_3	359.42	32.70	35.65	33.73	48.79	92.54	0.09	1.49	**2.60**
An12g05700		RHA (α-rhamnosidase)	GH78	139.26	18.33	21.43	16.87	21.87	60.97	0.12	1.19	**2.85**
An12g10390	*faeB*	FAE (feruloyl esterase)	Esterase	228.30	29.91	59.77	34.99	62.01	49.20	0.15	**2.07**	0.82
An04g09070		RHA (α-rhamnosidase)	GH78	161.69	36.46	34.26	37.26	34.24	58.43	0.23	0.94	1.71
An19g00270	*pelD*	PEL (pectin lyase)	PL1_4	58.52	27.49	16.91	13.77	102.58	13.01	0.24	**3.73**	0.77
An14g01130	*rglA*	RGL (rhamnogalacturonan lyase)	PL4_1	65.30	36.72	24.95	17.01	18.73	162.52	0.26	0.51	**6.52**
An03g06310	*pmeA*	PME (pectin methyl esterase)	CE8	111.27	30.14	40.10	31.79	163.56	27.60	0.29	**5.43**	0.69
An02g10550	*abnC*	ABN (endo-arabinanase)	GH43	640.03	1,143.41	1,323.94	201.91	613.33	2,662.51	0.32	0.54	**2.01**
An11g04040	*pgxA*	PGX (exo-polygalacturonase)	GH28	53.88	28.03	38.30	18.36	31.43	20.73	0.34	1.12	0.54
An01g06620		RHA (α-rhamnosidase)	GH78	143.12	63.40	88.79	54.59	79.08	157.73	0.38	1.25	1.78
An05g02440	*pgaC*	PGA (endo-polygalacturonase)	GH28	51.81	45.85	69.56	22.90	105.27	235.82	0.44	**2.30**	**3.39**
An03g02080	*rgxB*	RGX (exo-rhamnogalacturonase)	GH28	21.05	11.86	11.32	11.33	12.23	64.99	0.54	1.03	**5.74**
An18g04100		EXG (exo-1,3-galactanase)	GH5_9	75.73	318.09	94.55	41.80	242.62	1,311.78	0.55	0.76	**13.87**
An15g03550		ABN (endo-arabinanase)	GH43	38.22	18.07	19.97	22.21	21.55	19.85	0.58	1.19	0.99
An14g05820	*lacE*	LAC (β-1,4-galactosidase)	GH35	36.41	20.28	24.98	21.55	30.75	94.55	0.59	1.52	**3.78**
An15g07160	*pelF*	PEL (pectin lyase)	PL1_4	153.79	218.71	247.77	95.98	184.79	157.30	0.62	0.84	0.63
An09g02160	*rgaeA*	RGAE (rhamnogalacturonan acetyl esterase)	CE12	18.83	18.73	31.90	16.09	25.90	79.74	0.85	1.38	**2.50**
An07g04930		ABN (endo-arabinanase)	GH43	32.24	41.47	37.23	29.68	32.65	33.61	0.92	0.79	0.90
An03g00190	*pelB*	PEL (pectin lyase)	PL1_4	42.55	23.29	22.61	39.42	69.50	24.56	0.93	**2.98**	1.09
An09g00880	*abfD*	ABF (α-arabinofuranosidase)	GH51	22.20	20.62	18.67	20.70	21.25	17.33	0.93	1.03	0.93
An02g01400	*abnB*	ABN (endo-arabinanase)	GH43	47.12	38.38	51.70	44.40	38.51	47.14	0.94	1.00	0.91
An12g02550	*faeC*	FAE (feruloyl esterase)	CE1	13.62	12.94	13.22	13.15	15.91	438.93	0.97	1.23	**33.20**
An01g14650	*rgxA*	RGX (exo-rhamnogalacturonase)	GH28	33.98	30.07	34.52	32.86	40.42	97.71	0.97	1.34	**2.83**
An18g04800		RHA (α-rhamnosidase)	GH78	37.90	47.60	59.57	37.08	50.33	28.91	0.98	1.06	0.49
An01g14670	*pgaE*	PGA (endo-polygalacturonase)	GH28	16.76	71.48	813.08	16.56	42.26	54.63	0.99	0.59	0.07
An07g04420	*lacD*	LAC (β-1,4-galactosidase)	GH35	20.57	18.70	18.04	20.39	18.40	19.68	0.99	0.98	1.09
An09g05350		FAE (feruloyl esterase)	Esterase	55.07	175.65	260.91	55.16	156.40	48.86	1.00	0.89	0.19
An10g00870	*plyA*	PLY (pectate lyase)	PL1_7	23.10	25.39	19.17	23.26	21.65	16.87	1.01	0.85	0.88
An14g05340	*urhgB*	URH (unsaturated rhamnogalacturonyl hydrolase)	GH105	12.06	11.92	14.88	12.65	12.15	22.93	1.05	1.02	1.54
An11g08700	*rhgE*	RHG (endo-rhamnogalacturonase)	GH28	24.26	27.90	24.96	26.05	28.55	23.50	1.07	1.02	0.94
An04g02780		FAE (feruloyl esterase)	Esterase	22.74	22.76	23.23	24.76	27.51	23.02	1.09	1.21	0.99
An06g02070	*rhgC*	RHG (endo-rhamnogalacturonase)	GH28	17.77	16.16	16.47	20.66	19.94	25.24	1.16	1.23	1.53
An16g06990	*pgaA*	PGA (endo-polygalacturonase)	GH28	15.76	25.25	21.40	18.47	30.92	20.20	1.17	1.22	0.94
An15g05370	*pgaII*	PGA (endo-polygalacturonase)	GH28	12.91	15.17	31.67	15.25	275.07	834.09	1.18	**18.13**	**26.34**
An08g01100		EXG (exo-1,3-galactanase)	GH5_23	101.13	128.96	149.26	124.33	181.78	198.31	1.23	1.41	1.33
An12g00950	*rhgA*	RHG (endo-rhamnogalacturonase)	GH28	18.80	30.06	26.87	23.65	24.93	54.44	1.26	0.83	**2.03**
An11g07660	*exgD*	EXG (exo-1,3-galactanase)	GH5_9	110.74	105.88	119.10	152.14	153.63	104.04	1.37	1.45	0.87
An02g04900	*pgaB*	PGA (endo-polygalacturonase)	GH28	27.96	28.38	30.44	39.77	249.39	120.72	1.42	**8.79**	**3.97**
An16g02730	*abnD*	ABN (endo-arabinanase)	GH43	18.26	22.26	39.49	32.11	52.08	36.39	1.76	**2.34**	0.92
An11g04030	*pelC*	PEL (pectin lyase)	PL1_4	13.55	13.87	13.94	26.70	14.21	12.45	1.97	1.02	0.89
An14g04200	*rhgB*	RHG (endo-rhamnogalacturonase)	GH28	3,356.80	5,671.34	6,516.57	7,068.13	5,647.22	6,376.02	**2.11**	1.00	0.98

a Differences in gene expression values between the two culture conditions are illustrated by the fold change between the 1% sugar beet pulp (SBP) culture compared to the 1% polygalacturonic acid (PGA) culture at each time point. Values for upregulated genes (>2-fold values) are indicated in boldface, while values for downregulated genes (<0.5-fold values) are underlined. The complete set of CAZyme-encoding genes is described in Data Set S4.

**TABLE 2:** Comparative analysis of the expression of metabolic genes involved in metabolism of d-galacturonic acid, l-rhamnose, or l-arabinose and d-xylose in A. niger liquid cultures with 1% sugar beet pulp or 1% polygalacturonic acid

Gene ID	Gene name	Metabolic pathway	Expression with growth on:						Fold change in expression^*a*^		
			PGA			SBP					
			2 h	8 h	24 h	2 h	8 h	24 h	2 h	8 h	24 h
An02g07710	*gaaA*	d-Galacturonic acid	6,054.20	4,584.99	4,704.71	48.23	1,415.94	451.26	0.01	0.31	0.10
An16g05390	*gaaB*	d-Galacturonic acid	9,971.13	1,1264.16	11,400.66	118.85	4,623.06	528.18	0.01	0.41	0.05
An02g07720	*gaaC*	d-Galacturonic acid	9,738.10	9,762.59	9,536.53	120.25	2,604.60	870.24	0.01	0.27	0.09
An16g04770	*garB*	d-Galacturonic acid	905.68	269.06	240.27	151.64	138.75	159.85	0.17	0.52	0.67
An11g01120	*larA*	PCP/d-galacturonic acid	8,004.89	5,888.88	6,209.71	738.05	7,335.86	877.23	0.09	1.25	0.14
An08g01740	*xyrB*	PCP	1,661.46	100.20	118.87	152.42	590.32	391.79	0.09	**5.89**	**3.30**
An01g10920	*ladA*	PCP	5,723.10	77.96	111.08	611.80	2,149.41	247.24	0.11	**27.57**	**2.23**
An08g01930	*lxrA*	PCP	4,773.27	39.21	163.31	407.68	1,199.78	86.13	0.09	**30.60**	0.53
An07g03570	*lxrB*	PCP	1,711.10	619.79	418.27	1,399.26	521.25	97.90	0.82	0.84	0.23
An01g03740	*xyrA*	PCP	6,343.96	82.24	305.83	320.66	1,213.81	3,287.31	0.05	**14.76**	**10.75**
An12g00030	*xdhA*	PCP	11,037.45	317.80	569.39	630.74	2,243.42	2,139.00	0.06	**7.06**	**3.76**
An07g03140	*xkiA*	PCP	1,772.38	62.07	81.64	165.47	939.56	370.55	0.09	**15.14**	**4.54**
An13g00930	*lraA*	l-Rhamnose pathway	1,059.12	23.84	28.75	25.71	42.16	84.55	0.02	1.77	**2.94**
An05g02050		l-Rhamnose pathway	107.54	16.83	19.69	17.02	21.12	36.58	0.16	1.25	1.86
An13g00940	*lrlA*	l-Rhamnose pathway	279.58	91.58	142.10	90.66	92.12	117.99	0.32	1.01	0.83
An13g00920	*lrdA*	l-Rhamnose pathway	3,778.54	62.01	152.31	29.79	177.14	412.13	0.01	**2.86**	**2.71**
An03g02490	*lkaA*	l-Rhamnose pathway	888.02	340.30	522.12	77.28	337.28	396.47	0.09	0.99	0.76
An12g05070		l-Rhamnose pathway	69.96	34.36	28.34	33.50	24.88	30.25	0.48	0.72	1.07

a Differences in gene expression values between the two culture conditions are illustrated by the fold change between the 1% sugar beet pulp (SBP) culture compared to the 1% polygalacturonic acid (PGA) culture at each time point. Upregulated genes (>2-fold values) are indicated in boldface, while downregulated genes (<0.5-fold values) are underlined. The complete set of metabolic genes is described in Data Set S5 in the supplemental material.

In contrast, the 8-h samples showed upregulation of 24 genes and downregulation of only one polygalacturonase gene (*pgxB*) involved in pectin degradation in the SBP culture compared to the PGA culture (Table 1). Interestingly, the *pgxB* gene was the only gene involved in pectin degradation that showed significant downregulation in the SBP culture compared to the PGA culture at each time point. On a metabolic level, the levels of expression of l-rhamnose catabolic genes were comparable between the two conditions after 8 h, except for *lrdA*, which showed a substantial increase in the SBP sample compared to the 2-h time point (Table 2). The expression of *lrdA* increased 2.9-fold in the SBP sample compared to the PGA culture after 8 h. On the other hand, the expression of PCP genes was highly upregulated in the 8-h SBP culture compared to the 2-h time point (Table 2), which can be associated with the increased expression of arabinofuranosidase (*abfA*, *abfB*, and *abfC*) and endo-arabinanase (*abnA*, *abnC*, *abnD*) genes (Table 1), as well as increased expression of genes involved in (arabino)xylan (*axhA*, *xlnC*/*xynA*, *xlnD*/*xynD*, *xynA*, *xlnB*/*xynB*, *xarB*, *gbgA*), or xyloglucan (*xegA*, *axlA*/*xylS*) degradation (see Data Set S4 in the supplemental material). The actions of their corresponding enzymes result in release of l-arabinose and d-xylose, which are metabolized through the PCP.

The 24-h samples showed upregulation of 19 and downregulation of 13 pectinolytic genes in the SBP culture compared to the PGA culture (Table 1). Two feruloyl esterase genes (*faeA*, *faeC*) were significantly upregulated in the SBP culture compared to the PGA culture at this time point. Feruloyl esterases act as accessory enzymes in the degradation of hemicellulose [e.g., (arabino)xylan] and pectin (32). However, the 24-h SBP culture showed predominant expression of (arabino)xylanolytic, xyloglucanolytic, and cellulolytic genes (Data Set S4). At a metabolic level, the expression of d-galacturonic acid catabolic genes in the 24-h SBP culture indicated a similar pattern to that of the 2- and 8-h samples, showing consistently lower expression values than the PGA cultures at all time points (Table 2). In contrast, the PCP genes showed significantly increased expression compared to the PGA cultures (Table 2). This increase of expression can be attributed to the degradation of xyloglucan and possibly residual (arabino)xylan, as previously indicated.

This comparative analysis shows that A. niger responds to the presence of pectin with the upregulation of major pectinolytic genes at an early stage of growth and shows a consistently high expression of d-galacturonic acid catabolic genes. However, due to the complex composition of SBP, the degradation of pectin is delayed, and both the degradation and utilization of pectin and pectin-derived compounds found in SBP occur at a later stage of growth, showing the adaptation to different substrates or substrate availability over time.

### A. niger liquid sugar beet pulp cultures show higher differential gene expression than solid cultures.

Whether, and if so to what extent, submerged cultures resemble growth conditions in solid medium has not been addressed in filamentous fungi. In this study, we compared the gene expression profiles of liquid SBP after 2, 8, and 24 h to those on solid SBP plates. Sampling time points for the liquid cultivation were based on previous experiments to match first, intermediate, and late responses, as would also be present in the colony peripheral, middle, and central zones. In these analyses, the gene expression patterns were studied in different parts of the colony: rings 1 and 2 located at the center of the colony, ring 3 at the intermediate part of the colony, and rings 4 and 5 being the outer (and younger) rings of the colony, as previously reported (8). The separation of a mycelial colony into five concentric rings allows the comparison of the young mycelium exploring fresh SBP (rings 4 to 5) with the fungus growing in liquid medium at early time points (2-h SBP). Similarly, this experimental setup allows the comparison of the gene expression pattern of the older mycelium growing on (partially) utilized SBP (rings 1 to 3) with that of the fungus grown in liquid medium at latter time points (24-h SBP).

A PCA of duplicate samples showed that the overall gene expression pattern after 2, 8, and 24 h of growth in liquid SBP can be clearly distinguished into three distinct groups (see Fig. S3, orange cluster, in the supplemental material), indicating the adaptation of the fungus to the substrate composition over time. In addition, the A. niger transcriptomic response in liquid SBP is clearly different from that on solid SBP, since two distinct clusters were observed in the PCA analysis (Fig. S3, orange and green clusters). The transcriptomic responses among rings 1 to 4 were relatively similar, while the outer ring of the colony (ring 5) showed a more different profile. For liquid cultures, the 24-h SBP samples were most distinct, while SBP ring 5 and SBP at 8 h were the most comparable samples between solid and liquid cultures, respectively.

**FIG 4:**
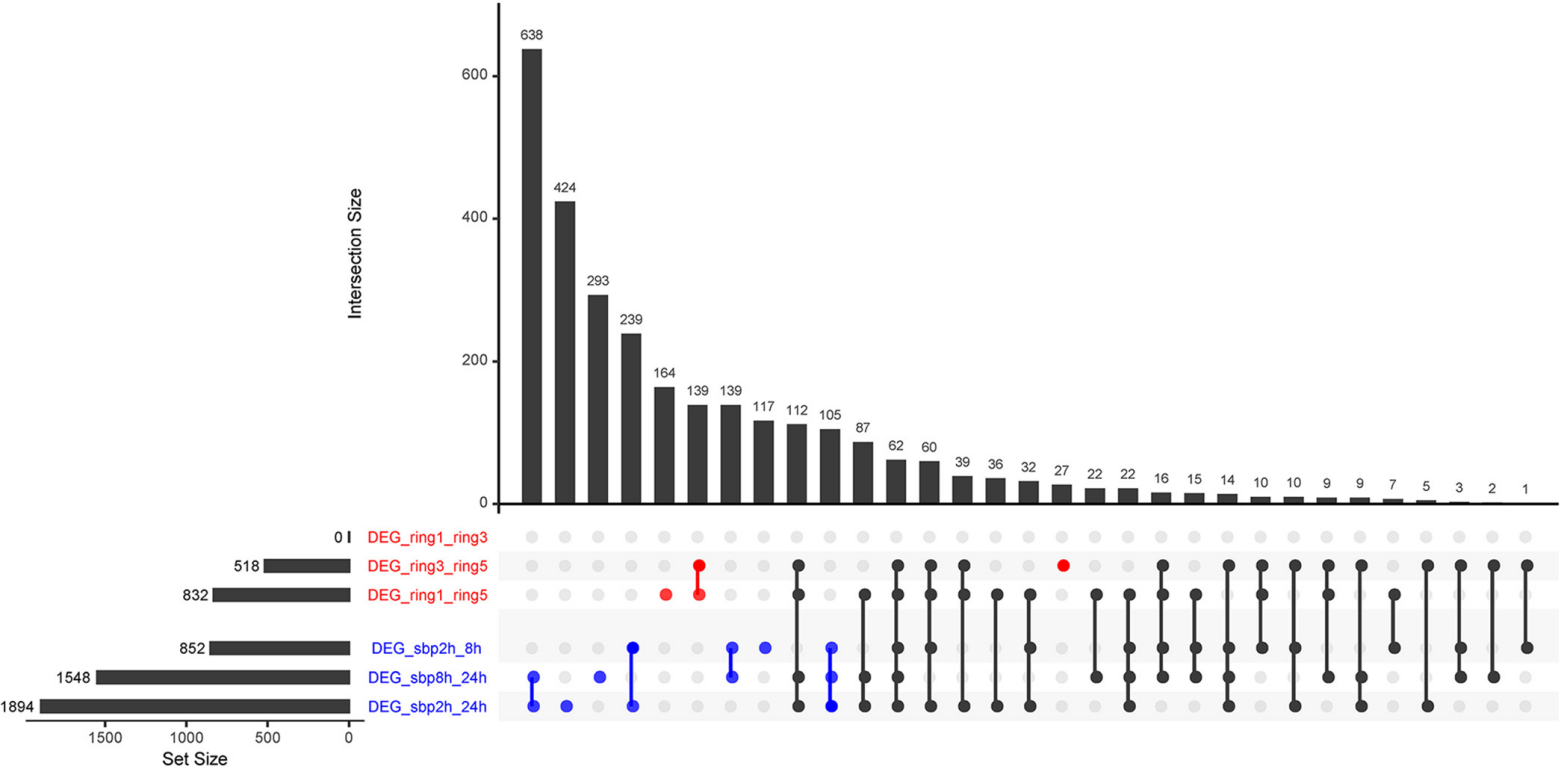
Differentially expressed genes (DEGs) in A. niger in response to sugar beet pulp solid and submerged cultures.

Analysis of differentially expressed genes (DEGs) between solid and liquid SBP cultures showed that gene expression variability within the fungal colony in solid plates was much lower than that of liquid cultures (Fig. 4). These results together with the PCA already indicate that the genetic response to a substrate is strongly affected by the culture conditions. Regarding solid medium, there was a positive correlation between the physical distance among the rings and the number of DEGs, as previously reported (8). No DEGs were found between rings 1 and 3 (Fig. 4), which are relatively close within the colony. In contrast, 518 DEGs were found between rings 3 and 5, 27 of which were unique DEGs under these conditions. Furthermore, 832 DEGs were identified between rings 1 and 5, 164 of which were unique DEGs between these conditions (Fig. 4).

Liquid SBP cultures showed the highest overall number of (distinct) genes that were differentially expressed over time, with a positive correlation between growth time and the number of DEGs. A total of 852 DEGs were found in A. niger grown in liquid SBP after 2 and 8 h, of which 139 were unique DEGs between these two time points. Between 8 and 24 h of growth in liquid SBP, 1,548 DEGs were identified, with 293 unique DEGs between both culture conditions. In addition, 1,894 DEGs were found in 2-h liquid SBP cultures compared to 24-h liquid SBP cultures. The liquid culture condition presented more than twice as many DEGs as the highest number of DEGs seen in the solid SBP samples (Fig. 4).

Comparisons of the DEGs between solid and liquid cultures showed that more than half (63.5%) of DEGs detected in comparison between solid conditions were also detected under liquid conditions (Fig. 4). These results indicate that despite the large number of DEGs detected under all the conditions tested, the genes affected between solid and liquid cultures were very similar. The differences in the numbers of DEGs between solid and liquid culture conditions could be attributed to exposure of the fungus to different environmental conditions, such as aeration, oxygen diffusion rate, osmolarity, viscosity, density of spore inoculum, growth type, or substrate availability, which vary a lot between solid and liquid culture conditions (3, 33).

### The gene expression patterns of CAZyme-encoding genes between solid and liquid sugar beet pulp cultures show limited correlation in A. niger.

Genes encoding plant polysaccharide-degrading enzymes, which include cellulases, hemicellulases, pectinases, inulinases, and starch-degrading enzymes, were differentially expressed in solid and liquid SBP cultures (Fig. 1).

Pectin is one of the most complex polysaccharides found in plant cell walls, and approximately 14 CAZy families are predicted to contain pectinolytic enzymes (27). In solid SBP cultures, the expression profiles of pectinolytic genes differed from the center (ring 1) to the periphery (ring 5) as previously discussed (8). Several pectinolytic genes, such as *pgaC*, *rgaeA*, *abnA*, *pgxA*, and *pgxB*, showed higher expression at the center of the colony (rings 1 and 2) (Fig. 1), correlating with their highest expression values after 8 and 24 h in liquid SBP. However, the expression levels for these genes in liquid cultures were overall much lower than on solid cultures. Other pectinolytic genes, such as *pgaA*, *pgaB*, and *pgaII*, showed higher expression at the periphery of the colony (Fig. 1), which is in continuous contact with fresh SBP. However, none of these genes was induced at early time points in liquid SBP cultures, and one of them, *pgaII*, showed its highest expression values only after 24 h of growth in liquid SBP, suggesting little correlation in the expression profiles of these genes between the liquid and solid conditions. Other pectin-related genes, such as *pgaX*, *rhgA*, *lacA*, *abnA*, and *pelA*, showed consistent expression throughout the colony on solid medium, but this was not the case for all of these genes over time in liquid SBP cultures (Fig. 1). For instance, *pgaX* and *rhgA* showed constant (but much lower) gene expression values in liquid SBP cultures. In contrast, *lacA*, *abnA*, and *pelA* showed their highest expression values after 8 h of growth in liquid SBP, with *abnA* showing much higher expression values in liquid than in solid SBP medium.

Regarding hemicellulose degradation, more than 23 CAZy families are involved in the decomposition of these complex polysaccharides (34). Many (arabino)xylanolytic genes (e.g., *gbgA*, *axhA*, *xynA*, *xynB*, *axeA*), and xyloglucanolytic genes (*axlA*, *xegA*, An03g05530, *eglA*) were highly and consistently expressed throughout the mycelium grown on solid plates (rings 1 to 5), while in liquid SBP, these displayed a time-dependent expression (Fig. 1). In this context, the (arabino)xylanolytic and xyloglucanolytic genes were overall expressed at low levels in 2-h SBP liquid cultures, and their expression increased over time, with 8- and 24 -h cultures showing the highest expression of hemicellulase-encoding genes (Fig. 1). These results indicate that in solid medium, hemicellulolytic genes are induced from the center of the colony to the periphery. In contrast, induction of hemicellulolytic genes in liquid SBP medium occurs at a later stage of growth, showing more variability in expression over time and indicating that the use of hemicellulose differs from solid to liquid cultures.

Cellulose is a recalcitrant polysaccharide composed of d-glucose units. Different enzymatic activities are needed for its degradation, including β-1,4-d-endoglucanase (EGL), β-1,4-d-glucosidase (BGL), cellobiohydrolase (CBH), and lytic polysaccharide mono-oxygenase (LPMO) activities (35). Several cellulolytic genes, such as *eglB* and *bglA*, were highly expressed throughout the colony when grown on solid medium (rings 1 to 5), while in liquid SBP, these genes showed their maximum expression values after 8 and 24 h, respectively (Fig. 1). Other cellulolytic genes (e.g., An03g05330, An02g09270, An06g02040, An04g08550, An08g08240) showed very similar expression patterns in solid and liquid SBP cultures, being expressed at low levels under all the conditions tested (Fig. 1). Additional cellulolytic genes, such as *cbhB*, *agdA*, An07g08950, An14g02670, and An08g05230, showed a differential gene expression pattern over the colony, being highly expressed in the center (ring 1) and expressed at low levels in the periphery (rings 4 and 5). These results correlate with the depletion of other (preferred) compounds in the older part of the colony (ring 1), which likely forces A. niger to use cellulose as a carbon source in this zone, while in the periphery, preferred carbon sources such as hemicelluloses or pectins are still available. These results on solid SBP cultures correlated with results obtained in liquid SBP cultures, in which the cellulase-encoding genes were highly expressed after 24 h of submerged growth on SBP, also probably in response to the depletion of preferred carbon sources. In general, liquid SBP culture samples from later time points show higher expression of cellulolytic genes than solid SBP in A. niger. This could be explained by a faster depletion of other preferred carbon sources in liquid medium compared to solid medium due to the higher availability of the (partially) dissolved substrates. This is reflected, for example, by the cellulolytic genes *bglM*, An12g04610, and An08g01760, which showed low expression throughout the colony and were highly expressed in 24-h liquid SBP samples (Fig. 1). In addition, the genes *cbhA*, *cbhB*, and *eglA* showed higher gene expression in the 24-h liquid SBP samples than in solid cultures. These results are of high interest for the industry, since submerged cultures are preferred over solid cultures for enzyme production due to a better management of aseptic conditions and process control (36).

Although starch is not a main component of SBP (20), several amylolytic genes were induced in both solid and liquid SBP cultures. The glucoamylase-encoding gene *glaA* showed high constitutive expression in both solid and liquid SBP cultures, whereas the genes *agdD*, *agdF*, *amyD*, and *amyF* showed constantly low expression under both conditions (Fig. 1). In contrast, the genes *agdA*, *agdB*, *agdE*, and *aamA* showed higher gene expression in the periphery of the colony, while their expression reached the highest values after 8 to 24 h of growth in liquid SBP. This result correlates with a potentially high cellulose degradation at that time point, and thus, d-glucose release, which could induce these starch-degrading enzymes mediated by the amylolytic regulator AmyR (21, 22).

Taking all these results together, we conclude that although there is a partial correlation between gene expression in liquid and solid SBP cultures regarding (hemi)cellulolytic genes, there is very limited correlation between the gene expression pattern of the most relevant pectinolytic and amylolytic genes between solid and liquid cultures of A. niger grown in SBP.

### The expression of sugar metabolic genes shows high correlation between solid and liquid sugar beet pulp cultures in A. niger.

On solid medium, ∼80% of the genes involved in central carbon metabolism are constantly expressed across the fungal colony, except for l-rhamnose catabolic genes, as previously discussed (8). When we compared the expression pattern of these genes between SBP solid and liquid cultures, we observed that there is high correlation between both conditions (Fig. 3).

l-Rhamnose is mainly present in rhamnogalacturonans I and II within pectin polymers, accounting for approximately 1.4% of the total sugar composition of the SBP used in this study (Table S1). In solid medium, the genes involved in the l-rhamnose catabolic pathway (e.g., *lraA*, *lrlA*, *lrdA*) showed an overall decreasing expression profile from the center (rings 1 and 2) to the periphery (ring 5) of the colony (Fig. 3). This may be explained by the delayed uptake of l-rhamnose by A. niger when other preferred monosaccharides are available (31), especially since the inducer of the l-rhamnose catabolic pathway is l-2-keto-3-deoxyrhamnonate, a metabolic conversion product of l-rhamnose (37). These results correlate with the ones observed in SBP submerged cultures. Genes *lraA*, *lrlA*, *lrdA*, and *lkaA* showed increased expression over time, with higher expression at the latest time point (24 h) (Fig. 3). Expression of the transcriptional regulator RhaR remained low but constant throughout the colony and in liquid cultures (Fig. 2).

Regarding d-galactose, which accounts for 6.5% of the total sugar composition of the SBP used in this study (Table S1), there are comparable expression profiles of d-galactose metabolic genes in solid and liquid cultures (Fig. 3). In addition, expression of the transcriptional regulator GalX, involved in the activation of genes responsible for d-galactose utilization (38), remained low but constant throughout the colony and in liquid cultures (Fig. 2).

l-Arabinose and d-xylose, which account for 29 and 2.3%, respectively, of the total sugar composition of the SBP used in this study (Table S1), are catabolized through the PCP and the PPP (25). Genes involved in the PCP and PPP are constantly expressed across the colony (Fig. 3), as previously described (8). Similarly, these two pathways are also constantly active over time in submerged SBP cultures, with overall higher expression values at 24 h of growth (Fig. 3). Exceptions are *ladA* and *lxrA*, involved in l-arabinose catabolism through the PCP, which are repressed after 24 h of growth in liquid cultures. These results may indicate a stronger induction of d-xylose catabolism at that stage of growth in liquid cultures compared to solid cultures.

In the case of d-galacturonic acid, accounting for 27% of the total sugar composition of SBP (Table S1), there was only partial correlation between gene expression patterns of the catabolic genes in solid and liquid cultures. While *gaaA*, *gaaB*, and *gaaC* involved in the first steps of d-galacturonic acid catabolic pathway were highly and constantly expressed throughout the colony, they are expressed at low levels after 2 h of growth in liquid SBP. However, their expression strongly increases after 8 h of growth in liquid medium and slightly decreases after 24 h (Fig. 3). Expression of the pectinolytic transcription factor-encoding gene *gaaR* remained low but constant under all conditions (Fig. 2), whereas the repressor-encoding gene *gaaX* showed much higher expression in solid cultures compared to liquid cultures (Fig. 2). In liquid cultures, *gaaX* showed the highest expression after 8 h of growth, likely as a response to GaaR induction at this time point, and expression slightly decreased after 24 h. In solid cultures, *gaaX* showed higher expression in the center of the colony (rings 1 and 2), and expression decreased at the periphery (ring 5), even though the d-galacturonic acid catabolic genes were highly and constantly expressed throughout the colony.

Finally, genes involved in glycolysis and the TCA cycle are overall constantly expressed within the colony and over time in submerged cultures (Fig. 3). However, several differences can be seen between both conditions, as in the case of the *pdcA* gene, which shows strong repression after 24 h of growth in liquid culture, or *acuF*, which is repressed in ring 5 of the colony (Fig. 3).

In conclusion, we demonstrate that the genetic response of A. niger to a substrate is affected when different growth and culture conditions (solid/liquid) are used, even though there is a temporary effect in both solid and liquid cultures. This study therefore demonstrates that interpretation of results obtained from liquid cultures in the context of the behavior of a fungus in its natural habitat has a high risk of error. Therefore, the experimental setup must be adapted to the final application of each study. Submerged cultures are suitable for applied studies in which protein/enzyme production needs to be emphasized. Nevertheless, solid cultures are a better option aimed at studying the response of a fungus to its natural environment, since the results obtained from submerged cultures only partially explain the natural behavior of a fungus in solid environments.

## MATERIALS AND METHODS

### Strains, media, and culture conditions.

The A. niger N402 (*cspA1*) strain (39) was used in this study and was grown at 30°C. For solid cultures, colonies were grown as a sandwiched culture (40) in the presence of 1% SBP as previously described (8). The sugar composition of SBP used in this study is shown in Table S1. For liquid cultures, mycelia were grown in 1% SBP cultures as previously described (41). Briefly, freshly harvested spores were pregrown overnight in 250 ml of complete medium (CM) (42) containing 2% d-fructose in a rotary shaker at 250 rpm. The mycelium was harvested by filtration through sterile cheesecloth and rinsed with minimal medium (MM) (42), and 1 g (wet weight) mycelium was transferred into 50 ml MM containing 1% SBP. Mycelial samples were taken by filtration after 2, 8, and 24 h of incubation. The samples were stored at −20°C until further processing.

### RNA isolation and microarray processing.

RNA isolation and microarray hybridization were performed as described previously (8). In brief, mycelia obtained from solid and liquid cultures were grinded using a microdismembrator (B. Braun). RNA was extracted using TRIzol reagent (Invitrogen) according to the manufacturer’s instructions. RNA was purified using a Nucleospin RNA cleanup kit (Macherey-Nagel GmBh & Co.), and the concentration was measured at *A*_260_. RNA quality was analyzed with Agilent 2100 bioanalyzer using an RNA6000 LabChip kit (Agilent Technology). Microarray hybridization using the Affymetrix GeneChips A. niger Genome Array was performed at GenomeScan (Leiden, The Netherlands). Samples were evaluated in biological duplicates.

### Transcriptomic analysis.

Microarray data were analyzed using the Bioconductor tool package version 2.8 (http://www.bioconductor.org/), together with homemade Perl (version 5.0) and Python (version 3.0) scripts. Probe intensities were normalized for background by the robust multiarray average (RMA) method (43) using the R statistical language and environment. This method makes use of only perfect match (PM) probes. The average value of each gene’s normalized expression was calculated for fungal samples from the same growth condition. These gene expression values were visualized with heat maps using R package “gplots,” with the complete-linkage clustering method and Euclidean distance. The expression values of individual genes visualized in Fig. 1 to 3 are found in Data Sets S1 to S3, respectively, in the supplemental material. Intersection groups, representing unique sets of genes identified only between intersected elements, were visualized using the UpSetR package in R. The Limma package (44) of R was used to discover the significantly expressed genes between two different growth conditions. A fold change of 2 and adjusted *P* value of 0.05 were used as cutoffs. Gene functional annotations were based on a previous study (41).

### Data availability.

The microarray data for fungus grown on solid SBP plates were obtained from a previous study (8). New microarray data generated in this study have been deposited in the GEO database (https://www.ncbi.nlm.nih.gov/geo/) under accession no. GSE175954 (https://www.ncbi.nlm.nih.gov/geo/query/acc.cgi?acc=GSE175954).
