# Supplementary material for: The Cultivation Method Affects the Transcriptomic Response of Aspergillus niger to Growth on Sugar Beet Pulp
Source: Microbiol Spectr. 2021 Aug 25;9(1):10.1128/spectrum.01064-21. doi: 10.1128/spectrum.01064-21 (PMC8552599; doi:10.1128/spectrum.01064-21)
Supplement: SUPPLEMENTAL FILE 2 — Supplemental material. Download SPECTRUM01064-21_Supp_2_seq4.pdf, PDF file, 0.8 MB [file spectrum01064-21_supp_2_seq4.pdf]

Supplementary information

Title

The cultuvation method affects the transcriptomic response of *Aspergillus niger* to growth on sugar beet pulp

Authors

Sandra Garrigues[a,§], Roland S. Kun[a], Mao Peng[a], Birgit S. Gruben[a,b], Isabelle Benoit Gelber [a,b,#], Miia Mäkelä[c], Ronald P. de Vries[a,b*]

Affiliations

[a]Fungal Physiology, Westerdijk Fungal Biodiversity Institute & Fungal Molecular Physiology, Utrecht University, Uppsalalaan 8, 3584 CT Utrecht, The Netherlands

[b]Microbiology, Utrecht University, Padualaan 8, 3584 CH Utrecht, The Netherlands

[c]Department of Microbiology, University of Helsinki, Viikinkaari 9, 00790 Helsinki, Finland

[§]Present address: Department of Biotechnology, Instituto de Agroquímica y Tecnología de Alimentos, Consejo Superior de Investigaciones Científicas, Paterna, Valencia, Spain

[#]Present address: Centre for Structural and Functional Genomics, Department of Biology, Concordia University, 7141 Rue Sherbrooke Ouest, Montréal, QC H4B 1R6, Canada

*Corresponding author: Ronald P. de Vries, r.devries@wi.knaw.nl

Table of contents

Figure S1. Principal component analysis (PCA) of gene expression profiles obtained from liquid sugar beet pulp (SBP) and polygalacturonic acid (PGA) cultures after 2, 8 and 24 h incubation.

Figure S2. Hierarchical clustering of CAZyme-encoding genes involved in pectin degradation in *A. niger* grown on 1% sugar beet pulp (SBP) or 1% polygalacturonic acid (PGA).

Figure S3. Principal component analysis (PCA) of gene expression profiles obtained from liquid sugar beet pulp (SBP 2h, 8h, 24h, in orange) and solid sugar beet pulp (rings 1-5, in green) cultures.

Table S1. Sugar composition of sugar beet pulp used in this study.

Table S2. Abbreviations of enzymatic activities presented in this study.

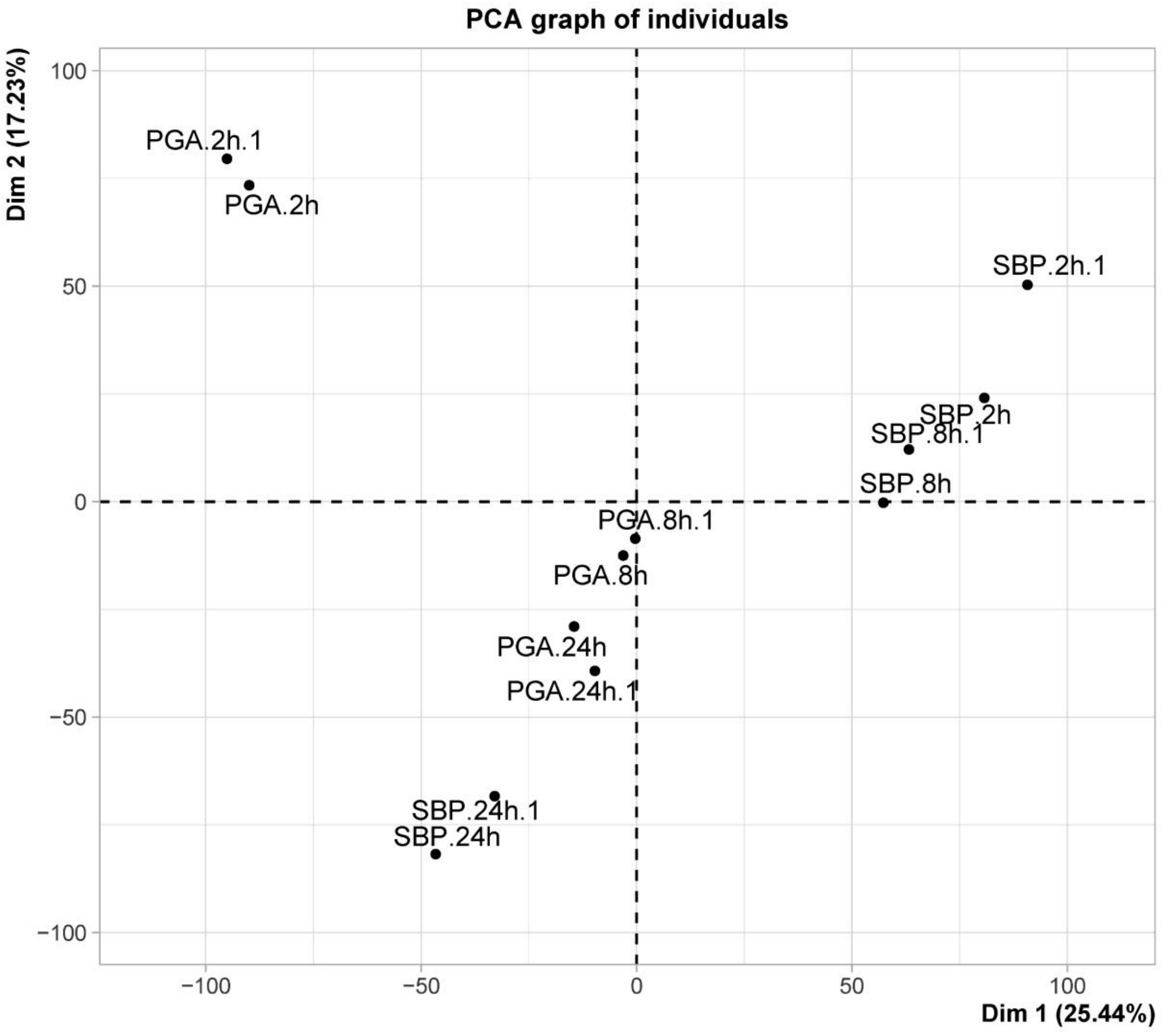

Figure S1: Principal component analysis (PCA) of gene expression profiles obtained from liquid sugar beet pulp (SBP) and polygalacturonic acid (PGA) cultures after 2, 8 and 24 h incubation. Each sphere represents the gene expression profile of an individual sample. Samples were analyzed in biological duplicates. The two axes accounted for 42.67% of variance.

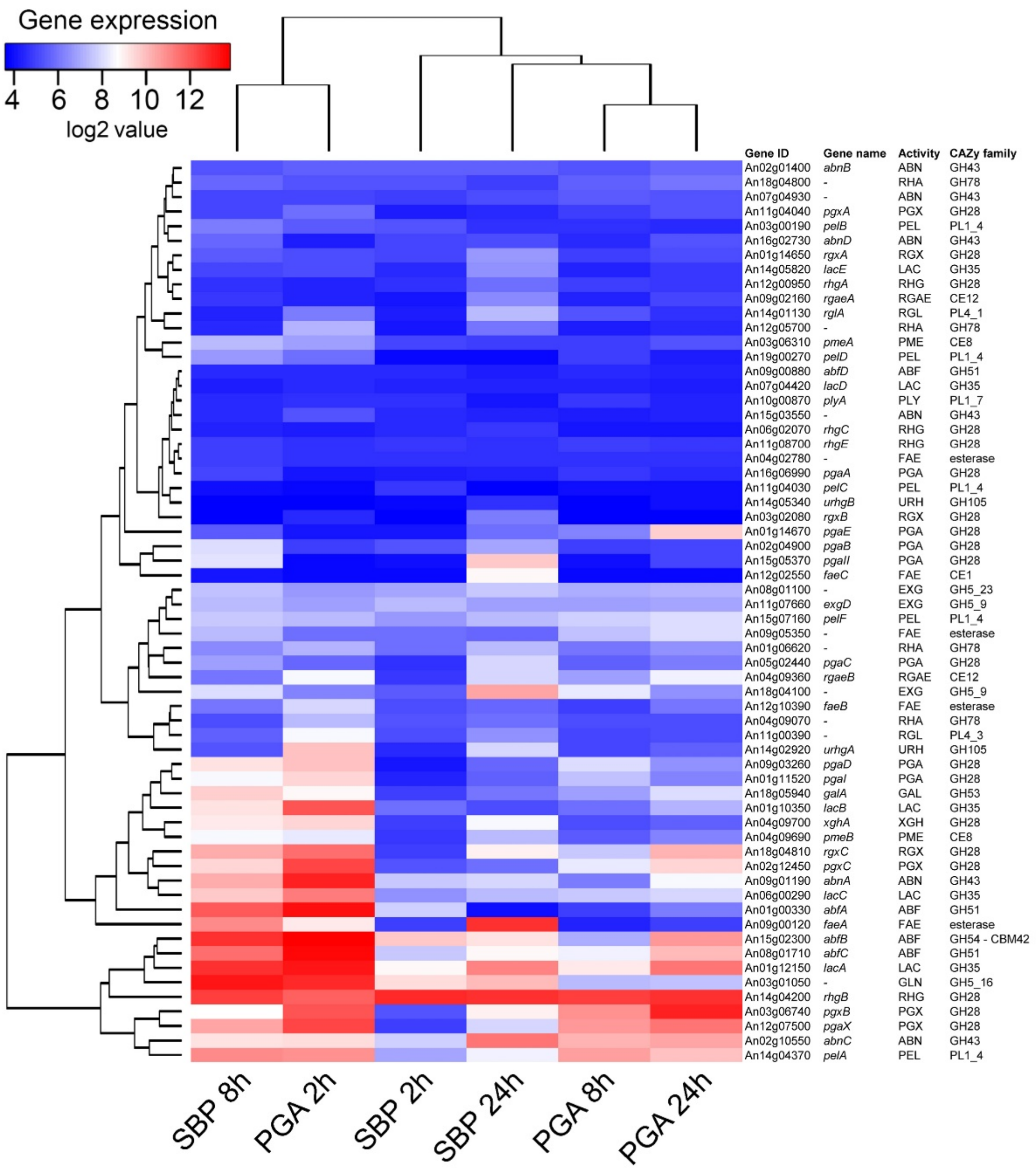

Figure S2: Hierarchical clustering of CAZyme-encoding genes involved in pectin degradation in *A. niger* grown on 1% sugar beet pulp (SBP) or 1% polygalacturonic acid (PGA). Gene expression data originate from 2, 8 and 24 h liquid cultures. Enzyme activity abbreviations are described in Table S2. Genes with an expression level < 20 across all samples were excluded from the analysis.

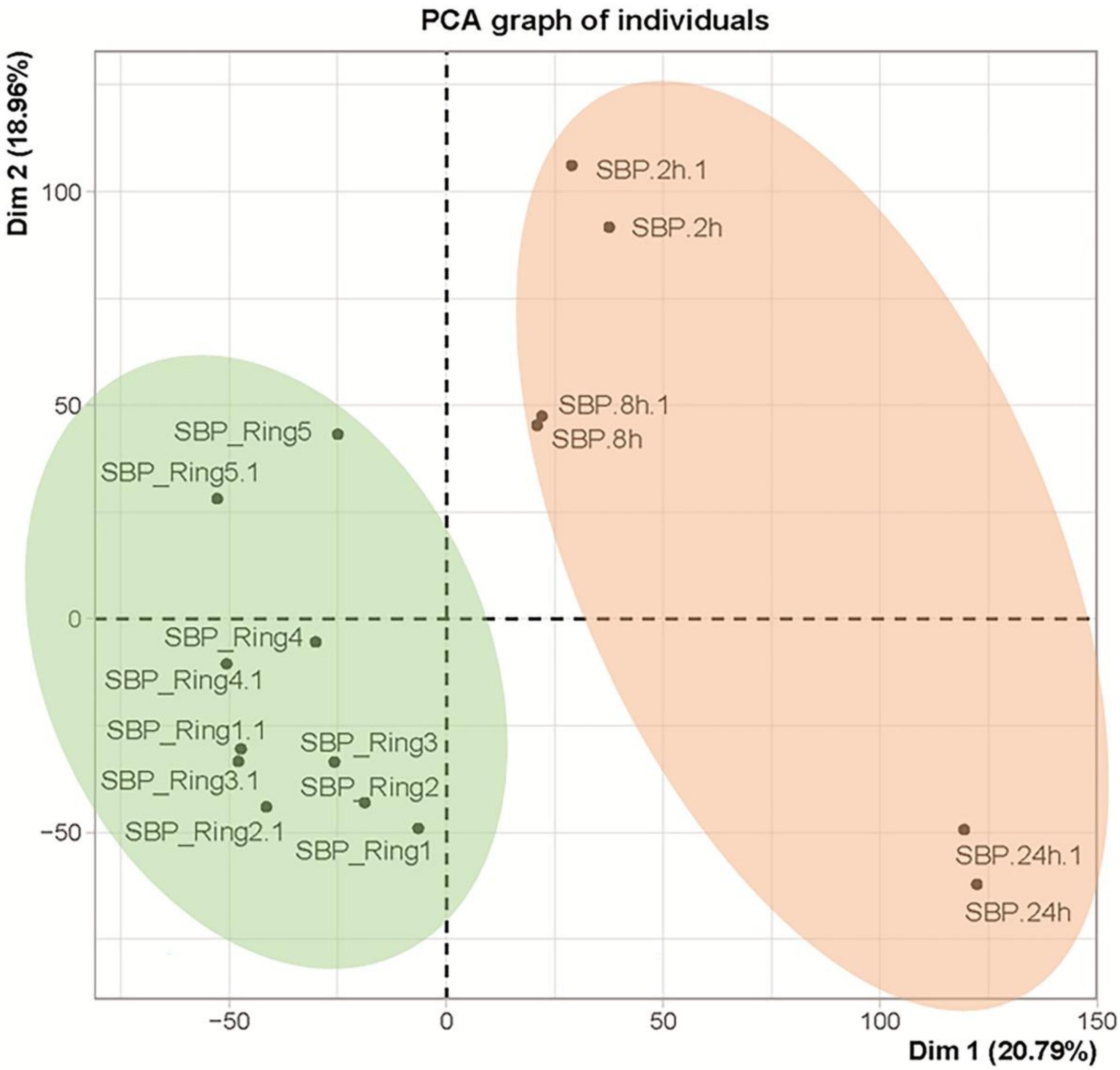

Figure S3: Principal component analysis (PCA) of gene expression profiles obtained from liquid sugar beet pulp (SBP 2h, 8h, 24h, in orange) and solid sugar beet pulp (rings 1-5, in green) cultures. Each sphere represents the gene expression profile of an individual sample. Samples were analyzed in biological duplicates. The two axes accounted for 39.75% of variance.

Table S1. Sugar composition of sugar beet pulp used in this study. The composition was determined as previously described (1).

mol%							w/w%
L-arabinose	D-xylose	D-mannose	D-galactose	D-galacturonic acid	L-rhamnose	D-glucose	Total sugar
29	2.3	2.1	6.5	27	1.4	32	56

1. Mäkelä MR, Bouzid O, Robl D, Post H, Peng M, Heck A, Altelaar M, de Vries RP. 2017. Cultivation of *Podospora anserina* on soybean hulls results in an efficient enzyme cocktail for plant biomass hydrolysis. New Biotechnol 37:162-171.

Table S2. Abbreviations of enzymatic activities presented in this study.

Abbreviation	Activity
ABF	α-arabinofuranosidase
ABN	endo-arabinanase
AE	acetyl esterase
AFC	α-L-fucosidase
AGD	α-glucosidase
AGL	α-1,4-galactosidase
AGU	α-glucuronidase
AMY	α-amylase
AXE	acetyl xylan esterase
AXH	arabinoxylan arabinofuranohydrolase
AXL	α-xylosidase
BGL	β-1,4-glucosidase
BXL	β-1,4-xylosidase
CBH	cellobiohydrolase
CDH	cellobiose dehydrogenase
EGL	β-1,4-endo-glucanase
EXG	exo-1,3-galactanase
FAE	feruloyl esterase
GAL	β-1,4-endo-galactanase
GLA	glucoamylase
GLN	exo-1,6-galactanase
GUS	β-glucuronidase
INU	endo-inulinase
INX	exo-inulinase
LAC	β-1,4-galactosidase
LPMO	lytic polysaccharide monooxygenase
MAN	β-1,4-endo-mannanase
ML-EGL	β-1,3/β-1,4-endoglucanase
MND	β-1,4-mannosidase
PEL	pectin lyase
PGA	endo-polygalacturonase
PGX	exo-polygalacturonase
PLY	pectate lyase
PME	pectin methyl esterase
RGAE	rhamnogalacturonan acetyl esterase
RGL	rhamnogalacturonan lyase
RGX	exo-rhamnogalacturonase
RHA	α-rhamnosidase
RHG	endo-rhamnogalacturonase
SUC	invertase/β-fructofuranosidase
URH	unsaturated rhamnogalacturonyl hydrolase
XG-EGL	xyloglucanase
XGH	xylogalacturonase
XLN	β-1,4-endo-xylanase
